# Tunable Narrowband Silicon-Based Thermal Emitter with Excellent High-Temperature Stability Fabricated by Lithography-Free Methods

**DOI:** 10.3390/nano11071814

**Published:** 2021-07-13

**Authors:** Guozhi Hou, Qingyuan Wang, Yu Zhu, Zhangbo Lu, Jun Xu, Kunji Chen

**Affiliations:** National Laboratory of Solid State Microstructures, School of Electronics Science and Engineering, Collaborative Innovation Center of Advanced Microstructures, Jiangsu Provincial Key Laboratory of Advanced Photonic and Electronic Materials, Nanjing University, Nanjing 210093, China; ihougz@163.com (G.H.); 161190087@smail.nju.edu.cn (Q.W.); MG20230064@smail.nju.edu.cn (Y.Z.); luzhangbo123@outlook.com (Z.L.); kjchen@nju.edu.cn (K.C.)

**Keywords:** narrowband thermal emitter, Tamm plasmon polaritons, wavelength selective, lithography-free, thermal stability

## Abstract

Thermal emitters with properties of wavelength-selective and narrowband have been highly sought after for a variety of potential applications due to their high energy efficiency in the mid-infrared spectral range. In this study, we theoretically and experimentally demonstrate the tunable narrowband thermal emitter based on fully planar Si-W-SiN/SiNO multilayer, which is realized by the excitation of Tamm plasmon polaritons between a W layer and a SiN/SiNO-distributed Bragg reflector. In conjunction with electromagnetic simulations by the FDTD method, the optimum structure design of the emitter is implemented by 2.5 periods of DBR structure, and the corresponding emitter exhibits the nearly perfect narrowband absorption performance at the resonance wavelength and suppressed absorption performance in long wave range. Additionally, the narrowband absorption peak is insensitive to polarization mode and has a considerable angular tolerance of incident light. Furthermore, the actual high-quality Si-W-SiN/SiNO emitters are fabricated through lithography-free methods including magnetron sputtering and PECVD technology. The experimental absorption spectra of optimized emitters are found to be in good agreement with the simulated absorption spectra, showing the tunable narrowband absorption with all peak values of over 95%. Remarkably, the fabricated Si-W-SiN/SiNO emitter presents excellent high-temperature stability for several heating/cooling cycles confirmed up to 1200 K in Ar ambient. This easy-to-fabricate and tunable narrowband refractory emitter paves the way for practical designs in various photonic and thermal applications, such as thermophotovoltaic and IR radiative heaters.

## 1. Introduction

The tunable narrowband thermal emitters, which can improve the conversion efficiency of heat energy converting to photon energy, have attracted abundant attention from researchers due to their potential applications, such as solar thermophotovoltaic (STPV) system [1,2,3,4], radiative cooling [5,6,7], infrared stealth [8,9,10], IR gas sensing, etc. [11,12,13]. Aimed for these applications, emitters with optimized narrowband emission are of vital importance. Certain natural lossy materials such as rare-earth ceramics exhibit ideal narrowband emission peaks at the cutoff wavelength, but the absorbing region and the bandwidth are fixed to the inherent material property without adjustability [14,15]. Correspondingly, various artificial nanostructures to realize the selective and narrowband thermal emission peak have been designed and investigated in recent years, including 1D metallic grating structures [16], 2D or 3D photonic crystals [4,17,18,19,20,21], metal-insulator–metal metamaterials [22,23,24], materials using tunable the refractive index [25,26,27], and nanocomposites based on surface plasmon polaritons [28,29,30,31]. However, the difficulties in nanostructure fabrication, such as large-area manufacturing, size control at nanoscale, and cost, seriously hinder their practical applications. In contrast, the planar multilayer structures based on surface-state resonances are the most promising candidates to achieve desired selective emission over a large area, which can mitigate the fabrication limitations of nanostructures [32,33,34,35,36,37].

Recently, among multilayer structures, Tamm plasmon polaritons (TPP)-based 1D structures have garnered increasing attention as thermal emitters [38,39,40,41,42]. The TPP surface waves exist at the interface between a metallic mirror and distributed Bragg reflector (DBR) and have a zero in-plane wave vector and strong energy confinement [43]. Properly designed TPP-based thermal emitter can exhibit the outstanding narrowband emission peak at target wavelength with low emission in the longwave band, making its application in the STPV system particularly prominent due to the reduced thermalization loss and sub-bandgap loss [2,4]. According to Wien’s law, when the thermal emitters are intended to emit in a shorter mid IR range for STPV application, the emitter has to be heated above 1000 K [44]. Therefore, refractory metals should be chosen as metallic mirrors in fabrication. Yang et al. designed the first thermal emitters based on conventional and modified TPP structure with a thin emitting layer of W on a Si/SiO_2_ DBR and Si/SiO_2_ DBR on different thick metals, yet the TPP peak based on refractory metal aimed at target wavelength exhibited only weak TPP peaks with peak value less than 60% [39,40]. Moreover, the thermal emitter with a thin metal layer outside possesses bad mechanical stability and refractory properties, and high background emission due to the absorption of the thin metal layer outside. Then, a TPP emitter composed of TiN and DBR with high-temperature stability and good peak value was proposed by Yang et al. [41]. However, as the simulation results show in Figure S1, the intrinsic optical property of TiN causes the emitter to have a high secondary peak in the longwave range, resulting in a large amount of radiant heat loss. Thus, it is still challenging to fabricate the actual large-scale thermal emitters with excellent properties of tunable narrowband emission and low radiant heat loss in the longwave range, which also maintain stable performance against high temperature for practical thermal devices.

In this work, we theoretically and experimentally present a TPP-based high temperature robust silicon-based thermal emitter composed of high-quality a-SiN_x_/a-SiN_y_O_z_ (SiN/SiNO) multilayer and metallic W film on polished Si substrate with the advantages of lithography-free and high throughput. The proposed Si-W-SiN/SiNO emitter achieves the excellent tunable narrowband optical absorption (i.e., emissivity) performance and effectively suppresses the absorption in the longwave range below 7 µm, which can realize the concentrated narrowband photo re-emission. In conjunction with electromagnetic simulations by the FDTD method, the optical properties and structure of the thermal emitter are demonstrated and well optimized systematically, and the emitter exhibits the functionalities of angle and polarization insensitivity. Furthermore, the actual Si-W-SiN/SiNO emitters are realized through magnetron sputtering and PECVD technology with easy-to-fabricate way and high film quality. The absorption spectra of fabricated emitters are in good agreement with simulation results. Additionally, the fabricated emitter shows great thermal stability performance, maintaining excellent narrowband absorption characteristics after 1200 K annealing heating/cooling treatment for a total of 8 h. This lithography-free and refractory Si-W-SiN/SiNO emitter is more practical, compared to other structures with 2D or 3D nanopatterns, and holds more potential for use in practical photonic and thermal devices.

## 2. Materials and Methods

Device Fabrication: The fabrication process of the Si-W-SiN/SiNO multilayer emitter contained magnetron sputtering and plasmon-enhanced chemical vapor deposition (PECVD) techniques. First, the planar Si substrate was cleaned according to RCA standard process. Then, a Ta layer with thickness of 20 nm was sputtered as adhesive layer on a cleaned Si substrate, followed by a W layer with a thickness of 150 nm sputtered as an opaque bottom metal layer. The sputtering condition was 80 W of power and 40 sccm of Ar flow, and the sputtering rates of Ta and W were about 0.33 and 0.21 nm/s. Finally, the SiN/SiNO multilayer film was deposited through the PECVD system. The a-SiN_x_ layer was deposited with a power of 10 W, a SiH_4_/NH_3_ mixed flow rate of 5/5 sccm, and the a-SiN_y_O_z_ layer was deposited with a power of 10 W, a SiH_4_/N_2_O mixed flow rate of 5/50 sccm. The butterfly valve was fully open during depositing films, and the growth temperature was 600 K. The deposited rate of a-SiN_x_ and a-SiN_y_O_z_ was approximately 0.21 and 0.73 nm/s, and the refractive index of a-SiN_x_ and a-SiN_y_O_z_ was approximately 2.94 and 1.51.

FDTD simulation: Electromagnetic simulations were performed by the finite-difference time-domain (FDTD) method. A broadband plane wave source from 0.3 to 7 µm was used for illumination. The boundary conditions of calculated models are periodic in x–y directions and perfectly matched layers in the z direction. The values of reflection and transmission were detected by two power detectors on the top and bottom of the model, and the magnetic field intensity distribution was calculated by field intensity monitor crossing the model structure. The condition of simulation temperature is 300 K. The dielectric constants of materials are obtained from Palik’s database.

Characterizations: The surface morphologies of samples were characterized through a scanning electron microscope (SEM, ZEISS Sigma) and atomic force microscope (AFM tapping mode, Nanoscope III-D, Bruker, Germany), and the energy-dispersive X-ray spectroscopy (EDS) data were measured by a detector (Oxford, X-act) in SEM. The crystal structure of metal was tested by X-ray diffraction (XRD, Bruker D8 Advance). The reflection spectra of our fabricated samples in the range of 220–2600 nm and 2600–7000 nm were measured in the form of percentages at room temperature by the ultraviolet–visible–near–infrared spectrophotometer (UV 3600, Shimazu, Japan) and the FT-IR photometer (iS50, Nicolet, America), respectively. The UV3600 spectrophotometer system is equipped with two light sources (D2 lamp and halogen lamp) and three photodetectors (PMT, InGaAs, and PdS detectors) inside. The FT-IR photometer is equipped with two light sources (halogen lamp and Polaris long-life infrared lamp) and three photodetectors (DLaTGS, DTGS, and MCT detectors) inside. Additionally, the UV 3600 spectrophotometer is equipped with an integrating sphere model (ISR-3100) coated with BaSO_4_, and the optical system diagram of it is shown in Figure S7. The absorption spectra were then calculated by formula A = 100%—R (T ~ 0), where A is the absorptivity, T is the transmissivity, and R is reflectivity. Correspondingly, according to Kirchhoff’s law of thermal radiation, the absorptivity is equal to the emissivity of a surface in thermodynamic equilibrium [39].

## 3. Results and Discussion

**Figure 1:**
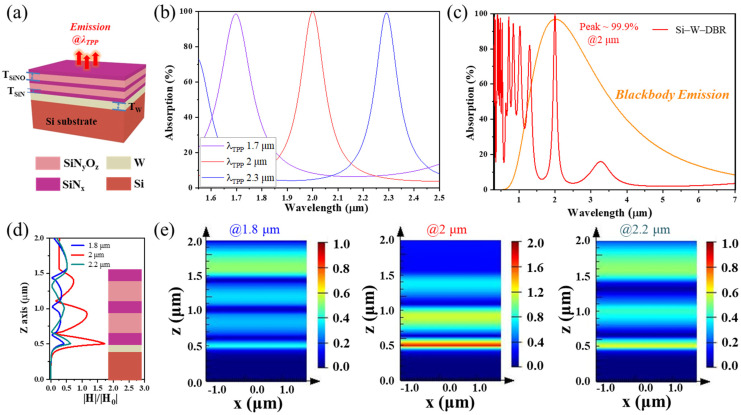
(**a**) Schematic diagram of a wavelength-selective and narrowband thermal emitter with Si-W-SiN/SiNO multilayers structure; (**b**) simulated absorption spectra of Si-W-SiN/SiNO emitter with TPP resonance peaks at 1.7, 2, and 2.3 µm, respectively. The wavelengths range from 1.5 to 2.4 µm; (**c**) simulated absorption spectra of Si-W-SiN/SiNO emitter with TPP resonance peak at 2 µm. The wavelengths range from 0.3 to 7 µm; (**d**,**e**) cross-sectional plot along the z axis of normalized magnetic field intensity distribution of the Si-W-SiN/SiNO structure at a wavelength of 1.8, 2.0, and 2.2 µm.

As schematically shown in Figure 1a, we proposed a wavelength-selective and narrowband emitter based on TPP with the basic structure composed of the one-dimensional SiN/SiNO photonic crystal and a metallic W film on Si substrate. As previously reported [43], the TPP mode can induce a narrowband absorption peak at its resonant wavelength, when it meets the condition of

(1) $$1-r_{W}\times r_{SiN/SiNO\;Phc}\sim 0,$$

where $r_{W}$ is the reflection coefficient of bottom metallic W film and $r_{SiN/SiNO\;Phc}$ is the reflection coefficient of a one-dimensional *SiN*/*SiNO* photonic crystal. Here, the optical properties of this proposed emitter were studied and optimized by the FDTD method, which was excited by a normal incident plane wave. As is depicted in Figure 1b, after achieving the optimized design, the Si-W-SiN/SiNO emitter exhibits an enhanced resonant excitation mode, with a tunable narrowband absorption (i.e., emissivity) peak at the wavelength of 1.7 µm, 2 µm and 2.3 µm, whose value is close to 100%. Additionally, for the emitter with the TPP resonance at 2 µm shown in Figure 1c, the absorption performance in the long-wavelength range is effectively suppressed with an average value of less than 5%. More information on each layer for this emitter is listed in Table S1. Correspondingly, the designed emission peak is consistent with the blackbody radiation peak at 1449 K, and the narrowband radiation can be efficiently carried out while the longwave thermal radiation loss can be reduced. To reveal the nature of resonance enhancement of excellent narrowband absorption performance, we investigated the distribution of magnetic fields inside the structure. Figure 1d,e shows the normalized magnetic field intensity values and distribution at a wavelength of 1.8 µm, 2.0 µm, 2.2 µm along the z axis. As can be observed, different from the distribution of magnetic field at 1.8 µm and 2.2 µm, a strongly localized magnetic field distribution exists at the junction of metallic W layer and SiN/SiNO photonic crystal at 2 µm, which is the TPP resonance wavelength. This localized magnetic field distribution indicates the existence of a strong optical Tamm state in the interface, and then the strong density of optical Tamm state in this interface enhances the decay rates and causes the localized energy of incident light to be completely dissipated by the W layer into heat energy. [45,46] Correspondingly, the Si-W-SiN/SiNO structure can exhibit an excellent optical absorption performance around the resonance wavelength. Additionally, the localized energy of incident light dissipated by the W film indicates that the W film is really acting as the thermal radiation source when operating at high temperatures.

**Figure 2:**
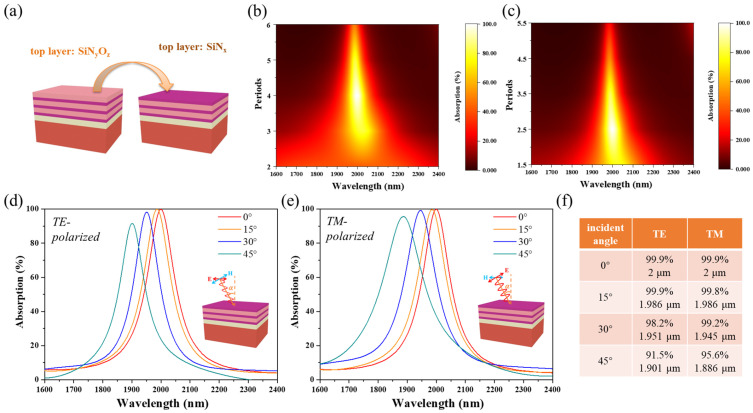
(**a**) Schematic diagrams of Si-W-SiN/SiNO emitters with different top layers. Simulated 2D absorption maps of Si-W-SiN/SiNO emitters with the absorption peak of TPP mode at 2 µm; (**b**) the number of DBR periods from 2 to 6 with the top layer of SiNO film; (**c**) the number of DBR periods from 1.5 to 5.5 with the top layer of SiN film. Simulated absorption spectra of Si-W-SiN/SiNO emitters with top SiN layer with the incident light angles from 0 to 45 for different polarization states; (**d**) with TE polarization; (**e**) with TM polarization; (**f**) the accurate resonance center value and position of TPP resonance peaks in (**d**,**e**).

To investigate the design consideration of the Si-W-SiN/SiNO emitters, various structures of the emitter with different periods of DBR structure were calculated through FDTD methods, as seen in Figure 2a. As shown in Figure 2b, the emitters composed of different periods of DBR structure and metallic W film can achieve varying degrees of enhanced narrowband absorption performance at TPP resonance wavelength. When increasing the number of DBR periods, the absorption peak gradually sharpened and the value of absorption peak increased, but when the number of DBR periods exceeded 4, the value of the absorption peak decreased significantly. The reason for this phenomenon is that the reflection effect of the DBR structure itself is not strong enough when the number of DBR periods is too small, and it cannot meet the Equation (1) and therefore hardly excites an excellent optical Tamm state with the bottom metallic W film. Furthermore, when the number of DBR periods is over 4, the reflection effect of the DBR structure becomes stronger so that less incident energy can be transmitted to the junction of metallic W film and DBR structure to be dissipated by the W film. Remarkably, when the number of DBR periods is no longer an integer, that is, by replacing the top layer with a high refractive index SiN film, the Si-W-SiN/SiNO emitter can obtain the optimal absorption performance with 2.5 periods of DBR structure as shown in Figure 2c. This can be attributed to the increased impedance mismatch with space when the outermost layer is a high refractive index film, resulting in a stronger reflection effect at a smaller number of DBR periods. Additionally, this reduces the growth process of three layers of film for Si-W-SiN/SiNO multilayer structures, reducing the overall thickness by approximately 800 nm, and contributes to a simpler fabrication process and higher temperature stability of actual emitters. Furthermore, as shown in Figure 2d,e, the narrowband absorption peak of the Si-W-SiN/SiNO emitter is not sensitive to polarization mode and has a considerable angular tolerance of incident light. It is due to the zero in-plane wave vector of Tamm plasmon, which means TPP mode can be excited by light propagating in free space. As shown in Figure 2f, while the incident angle increases to 45 degrees, the absorption peak shifts blue and the value of the absorption peak maintains above 90%. Meanwhile, the absorption peaks of TPP mode have little difference under TE and TM polarization states for the Si-W-SiN/SiNO emitter.

**Figure 3:**
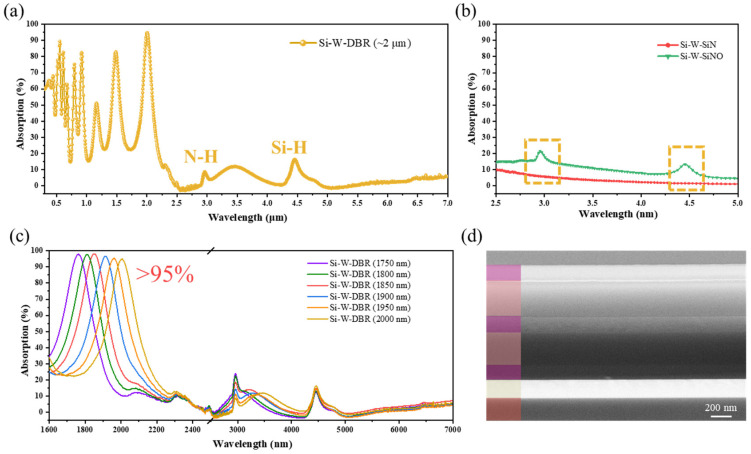
(**a**) Experimental optical absorption spectrum of Si-W-SiN/SiNO multilayer structure with TPP resonance peak at 2 µm; (**b**) experimental optical absorption spectra of Si-W-SiN and W-SiNO single-layer structure; (**c**) experimental optical absorption spectra of fabricated Si-W-SiN/SiNO multilayer structure with different TPP resonance peaks at 1750, 1800, 1850, 1900, 1950, and 2000 nm, respectively; (**d**) SEM characterization of fabricated Si-W-SiN/SiNO multilayer structure.

Based on the optimized simulation results, we fabricated the actual emitters with a high-quality Si-W-SiN/SiNO multilayer structure by magnetron sputtering and PECVD techniques. More experimental details are provided in the Experimental Section. Figure 3d shows the SEM cross section for the Si-W-SiN/SiNO emitter with TPP resonance peak at 2 µm. The metallic W film was 150 nm thick, which is enough to block light transmission and the value of transmissivity is approximately zero, and the thickness of the DBR structure using SiN and SiNO film was equal to the quarter optical path length of the targeted TPP resonance wavelength. It can be pointed out that although the agreement among the theoretical and experimental optical absorption spectra of Si-W-SiN/SiNO emitter, as shown in Figure 1b and Figure 3a, is fairly good, there are several discrepancies. Due to a large amount of hydrogen existing in the films prepared by PECVD methods [47,48], the vibration absorption peaks of the N-H bond and Si-H bond exist in the mid-infrared region of the experimentally prepared emitter. Correspondingly, the single film of SiN and SiNO layer were fabricated with the same technology. From the optical spectra of the mid-infrared region shown in Figure 3b, the vibration absorption peaks of the N-H bond and Si-H bond can be clearly seen in the SiNO layer, which is consistent with the peaks in the absorption spectrum of the Si-W-SiN/SiNO emitter.

Moreover, the optical property of the bottom metal is an important consideration that can affect the optical absorption spectra of the fabricated emitter. As depicted in Figure S3a,b, the lattice of as-sputtered W layer and W after 750 K annealing treatment were W (No.47-1319) with sharp peaks assigned to (200) and (210) planes. After annealing temperature increasing to 900 K, the lattice of W was changed to W (No.04-0806) with a sharp peak assigned to the (110) plane, in which the W layer had higher optical reflection performance. During the process of depositing the multilayer structure of the emitter by PECVD, we set the annealing temperature to 750 K and 900 K, respectively, so that the bottom W layer exhibited two different lattices. It can be inferred that when the lattice of tungsten is W (No.04-0806), the Si-W-SiN/SiNO emitter obtained shows a lower absorption performance in the longwave range, reducing the thermal radiation loss and closer to the simulated optical spectrum. Thus, the Si-W-SiN/SiNO emitters prepared later were all annealed at 900 K for 1 h. Furthermore, different Si-W-SiN/SiNO emitters with tunable TPP resonance peaks at 1750, 1800, 1850, 1900, 1950, and 2000 nm could be easily fabricated through an improved process, and the difference in these emitters is the thickness of the DBR structure. All emitters exhibited excellent narrowband absorption performance, with all peak values of emitters over 95%, as well as the well-suppressed absorption in longwave range, as depicted in Figure 3c. Additionally, calculated emissivity spectra of blackbody emitter and above different Si-W-SiN/SiNO emitters at 1449 K are shown in Figure S2. Compared to blackbody emitters, the Si-W-SiN/SiNO emitters exhibit obvious narrowband emission performance and suppressed emission loss in the longwave range.

**Figure 4:**
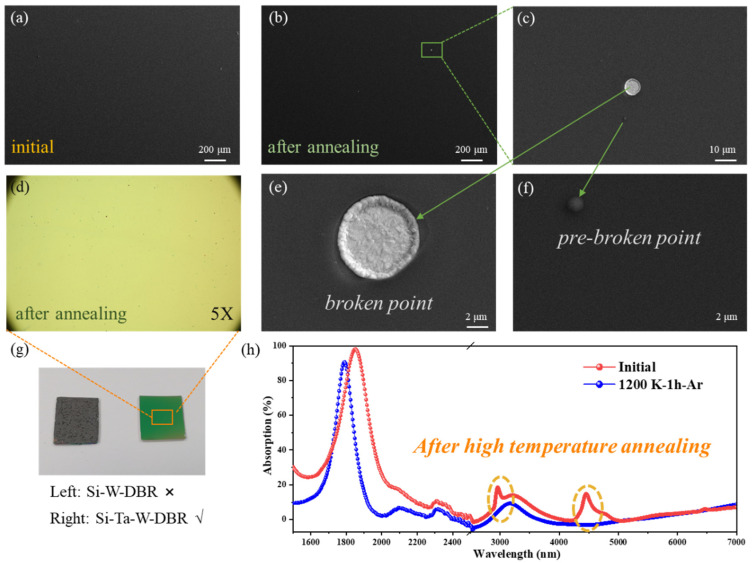
Morphology characterizations of Si-W-SiN/SiNO emitter before and after 1200 K annealing treatment; (**a**) SEM characterizations of initial Si-W-SiN/SiNO emitter with a magnification of 50; (**b**–**f**) optical microscopy and SEM characterizations of Si-W-SiN/SiNO emitter after 1200 K annealing for 1 h, with a magnification of 5, 50, 1000 and 5000, respectively; (**g**) digital photograph of Si-W-SiN/SiNO emitters after 1200 K annealing for 1 h with and without an adhesive layer; (**h**) experimental optical absorption spectra of fabricated Si-W-SiN/SiNO emitters before and after annealing at 1200 K in Ar atmosphere for 1 h.

It is worth noting that refractory properties are crucial for emitters to operate at high temperatures, such as STPV systems, where operating conditions may exceed 1100 K. Here, the annealing process via a furnace was conducted to investigate the refractory properties of the fabricated Si-W-SiN/SiNO emitter to test the suitability to extreme environments. The Si-W-SiN/SiNO emitter was annealed at 1200 K in Ar atmosphere for 1 h in a furnace. The emitter structure was seriously broken first due to the poor contact between W film and Si substrate. Thus, an adhesive layer of 20 nm Ta was sputtered on Si substrate before deposition of W layer to effectively improve the thermal stability of fabricated Si-W-SiN/SiNO emitter, as visually shown in Figure 4g and Figure S4. As can be seen from the measured spectra in Figure 4h, the optical properties of the Si-W-SiN/SiNO emitter had undergone little change but still maintained excellent narrowband optical absorption performance with a peak value of over 90%. Additionally, the absorption peak of TPP mode shifted blue and decreased slightly, and the vibration absorption peaks of the N-H bond and Si-H bond disappeared. The main reason for this change is due to hydrogen precipitation in the DBR multilayers at high temperatures, and the refractive index of SiN and SiNO layers changed where the structure could not meet the optimal conditions of TPP excitation. Correspondingly, detailed morphology characterizations of annealed Si-W-SiN/SiNO emitter were carried out, as depicted in Figure 4. As seen from the SEM characterization of low magnification in Figure 4a,b, the structure of the emitter remained intact after annealing, and there was no cracking phenomenon on the surface of the film so that the emitter could maintain good optical performance. Additionally, as the SEM and EDS characterizations in Figure 4b–f and Figure S5 there were very few small broken and pre-broken points on the surface of the sample, and the exposed metals at the bottom were oxidized due to the presence of even trace O_2_, which had little effect on optical performance. It is proved that the high-quality multilayer structure can effectively protect the underlying tungsten from oxidation. Moreover, the surface roughness of the samples was characterized by the AFM method, as shown in Figure S6. From the statistical roughness parameters in Table S2, the surface of fabricated layers through PECVD was quite smooth and intact where arithmetical average and root-mean-square roughness were all less than 2.1 nm, which contributed to the preparation of high-quality and stable emitters for use in high-temperature conditions.

**Figure 5:**
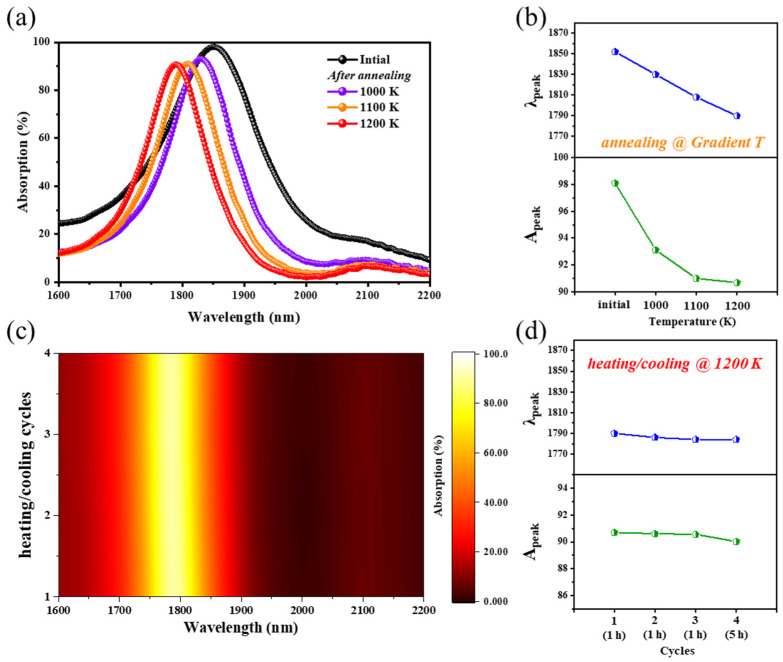
(**a**) Experimental optical absorption spectra of fabricated Si-W-SiN/SiNO emitter before and after annealing at gradient increasing temperature (1000 K, 1100 K, and 1200 K) in Ar atmosphere for 1 h; (**b**) the TPP resonance wavelength (λ_peak_) and the peak value (A_peak_) of experimental optical absorption spectra in (**a**); (**c**) experimental 2D absorption maps of fabricated Si-W-SiN/SiNO emitter after heating/cooling for 4 cycles. The first three cycles are annealed at 1200 K in Ar atmosphere for 1 h and the fourth cycle is annealed at 1200 K in Ar atmosphere for 5 h; (**d**) the TPP resonance wavelength (λ_peak_) and the peak value (A_peak_) of experimental optical absorption spectra in (**c**).

Furthermore, more high-temperature annealing tests were carried out to demonstrate the thermal stability of fabricated Si-W-SiN/SiNO emitter, such as gradient increasing temperature annealing and heating/cooling cycles for a longer time. As shown in Figure 5a,b, the absorption peak of TPP mode blueshifts from 1852 nm to 1784 nm when increasing the annealing temperature from 1000 K to 1200 K. Additionally, the peak value decreases rapidly at first and then slowly, decreasing by 4.974, 2.105, and 0.317 nm, respectively, at 1000 K, 1100 K and 1200 K. This can be attributed to the fact that the gradual increment in temperature leads to gradual hydrogen precipitation in the DBR multilayers and then affects the reflection effects of DBR structure. Additionally, heating/cooling cycle tests at 1200 K continued on this fabricated Si-W-SiN/SiNO emitter, and the relevant optical absorption spectra are depicted in Figure 5c,d. During high-temperature annealing cycle tests, the first three cycles were annealed at 1200 K in Ar atmosphere for 1 h, and the fourth cycle was annealed at 1200 K in Ar atmosphere for 5 h. Remarkably, due to the stable structure of the Si-W-SiN/SiNO emitter, the optical absorption performance maintains great consistency under the heating/cooling cycles of additional high-temperature annealing at 1200 K, even after a longer time of 5 h.

## 4. Conclusions

In summary, a wavelength-selective and narrowband thermal emitter based on a fully planar Si-W-SiN/SiNO multilayer with the advantages of simple structure and scalable large-scale fabrication was proposed with theoretical and experimental demonstration. The designed emitter achieves an excellent narrowband absorption performance in the expected wavelength with a peak value of nearly 100% while effectively suppressing the absorption in the longwave range. Combined with the FDTD calculation, it can be seen that by applying the SiN with a high refractive index as the top layer, the Si-W-SiN/SiNO multilayer emitter can inspire the outstanding absorption peak with fewer layers, which further simplifies the preparation process and improves the high-temperature stability. Additionally, the narrowband absorption peak is insensitive to polarization mode and has a considerable angular tolerance of incident light. Moreover, with the optimized fabrication technology of PECVD methods, the experimental absorption spectra of prepared high-quality emitters are found to be in good agreement with the simulated absorption spectra and exhibit the tunable narrowband absorption with all peak values of over 95%. Additionally, the fabricated emitter shows good resistance to ultra-high temperature, maintaining excellent narrowband absorption characteristics through up to 1200 K annealing treatment for heating/cooling cycles of a total of 8 h. Overall, we believe that the lithography-free and refractory Si-W-SiN/SiNO multilayer structure brings tremendous benefits and provides alternative candidates for various mid-IR applications, such as gas sensing, narrowband IR sources, and thermophotovoltaics.

## Supplementary Materials

The following are available online at https://www.mdpi.com/2079-4991/11/7/1814/s1, Figure S1: (a) The refractive index and extinction coefficient of TiN retrieved from Palik’s data in FDTD simulation; (b) the refractive index and extinction coefficient of W retrieved from Palik’s data in FDTD simulation; (c) simulated optical absorption spectra of Si-W-SiN/SiNO and Si-TiN-SiN/SiNO multilayer structure with TPP peak at 2000 nm, Figure S2: Calculated 2D emissivity map of blackbody emitter and fabricated Si-W-SiN/SiNO emitters with different TPP resonance peaks (1750, 1800, 1850, 1900, 1950, and 2000 nm) at 1449 K, Figure S3: The effect of different lattices of W on optical absorption performance, Figure S4: (a) Optical microscopy characterizations with five times magnification of initial Si-W-SiN/SiNO multilayer structure; (b) and (c) optical microscopy characterizations with five times magnification of Si-W-SiN/SiNO multilayer structure after 1200 K annealing for 1 h with and without adhesive layer, Figure S5: (a) Optical microscopy and SEM characterization of Si-W-SiN/SiNO multilayer structure after 1200 K annealing for 1 h, with a magnification of 5000; (b) and (c) EDS characterizations of the bare and intact area on Si-W-SiN/SiNO multilayer structure after 1200 K annealing for 1 h, Figure S6: AFM characterizations of different samples at 5 µm and 500 nm scale, Figure S7: Optical system diagram of the integrating sphere model (ISR-3100) in UV3600 systems, Table S1: The thickness and refractive index of Si-W-SiN/SiNO multilayer with TPP resonance peak at 2 μm, where *n* is the real part of the refractive index, and k is the imaginary part of the refractive index, Table S2: Statistical surface roughness parameters at 5 µm scale of Si-SiN single-layer structure, Si-SiNO single layer structure, Si-W single layer structure, initial Si-W-SiN/SiNO multilayer structure, and Si-W-SiN/SiNO multilayer structure after 1200 K annealing for 1 h.
