# Supplementary material for: Tunable Narrowband Silicon-Based Thermal Emitter with Excellent High-Temperature Stability Fabricated by Lithography-Free Methods
Source: Nanomaterials (Basel). 2021 Jul 13;11(7):1814. doi: 10.3390/nano11071814 (PMC8308295; doi:10.3390/nano11071814)
Supplement: Supplementary file 1 [file nanomaterials-11-01814-s001.zip › nanomaterials-1275025-SI.pdf]

Supplementary Materials

Tunable Narrowband Silicon-Based Thermal Emitter with Excellent High-Temperature Stability Fabricated by Lithography-Free Methods

Guozhi Hou, Qingyuan Wang, Yu Zhu, Zhangbo Lu, Jun Xu, and Kunji Chen

National Laboratory of Solid State Microstructures/School of Electronics Science and Engineering/Collaborative Innovation Centre of Advanced Microstructures/Jiangsu Provincial Key Laboratory of Advanced Photonic and Electronic Materials

Nanjing University, Nanjing, 210093, P. R. China

*Address correspondence to: junxu@nju.edu.cn.

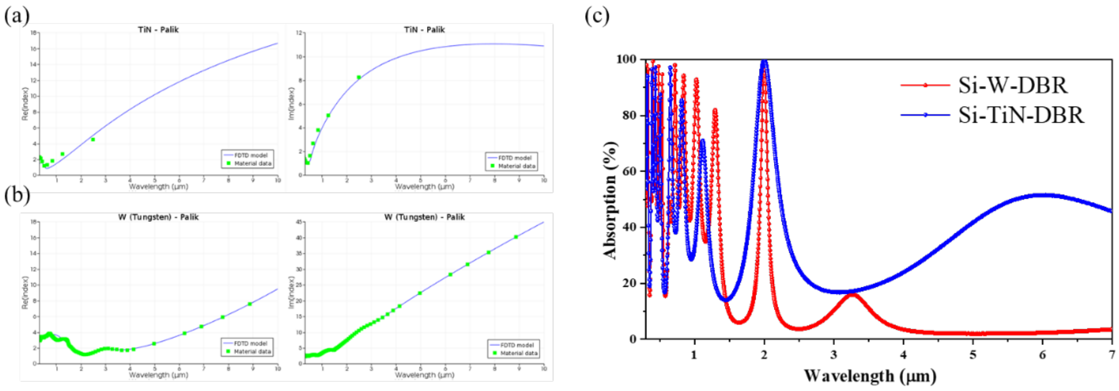

Figure S1. (**a**) The refractive index and extinction coefficient of TiN retrieved from Palik's data in FDTD simulation; (**b**) the refractive index and extinction coefficient of W retrieved from Palik's data in FDTD simulation; (**c**) simulated optical absorption spectra of Si-W-SiN/SiNO and Si-TiN-SiN/SiNO multilayer structure with TPP peak at 2000 nm.

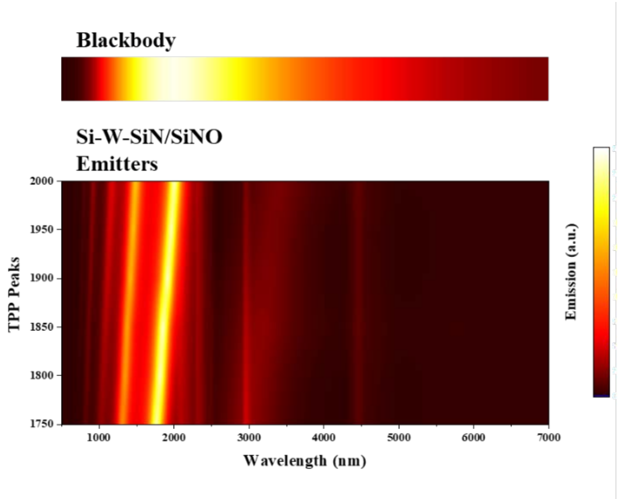

Figure S2. Calculated 2D emissivity map of blackbody emitter and fabricated Si-W-SiN/SiNO emitters with different TPP resonance peaks (1750, 1800, 1850, 1900, 1950, and 2000 nm) at 1449 K.

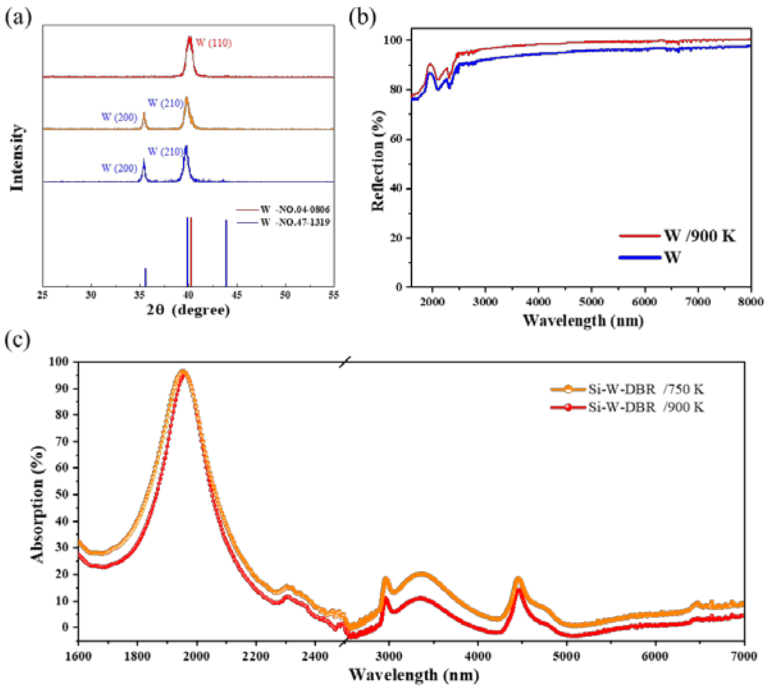

Figure S3. The effect of different lattice of W on optical absorption performance: (**a**) XRD spectra from bottom to top: as-sputtered W, W after 750 K heating treatment for 1h, W after 900 K heating treatment for 1h; (**b**) experimental optical reflection spectra of as-sputtered W and W after 900 K heating treatment for 1h; (c) experimental optical absorption spectra of fabricated Si-W-SiN/SiNO multilayer structure with TPP peak at 1950 nm, where the

temperature of annealing process is 750 K and 900 K, respectively.

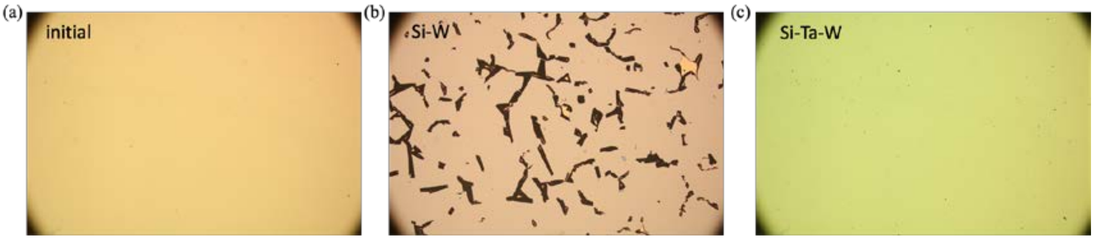

Figure S4. (**a**) Optical microscopy characterizations with 5 times magnification of initial Si-W-SiN/SiNO multilayer structure; (**b**) and (**c**) optical microscopy characterizations with five times magnification of Si-W-SiN/SiNO multilayer structure after 1200 K annealing for 1 h with and without an adhesive layer.

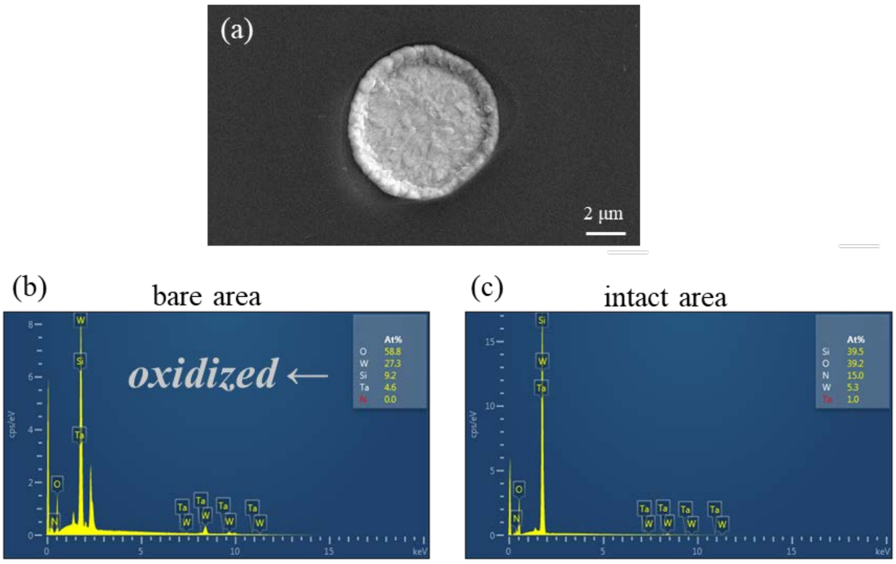

Figure S5. (**a**) Optical microscopy and SEM characterization of Si-W-SiN/SiNO multilayer structure after 1200 K annealing for 1 h, with a magnification of 5000 (**b**) and (**c**) EDS characterizations of the bare and intact area on Si-W-SiN/SiNO multilayer structure after 1200 K annealing for 1 h.

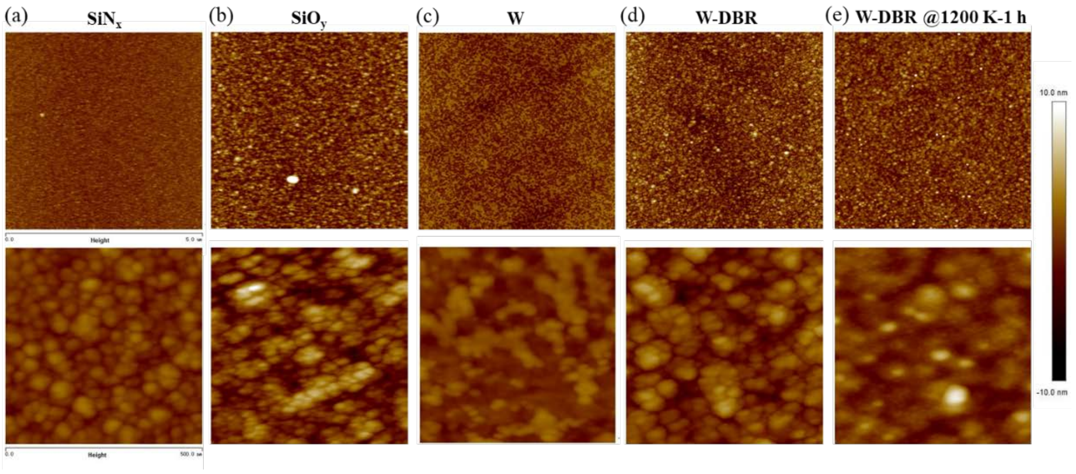

Figure S6. AFM characterizations of different samples at 5 μm and 500 nm scale: (**a**) Si-SiN single layer structure; (**b**) Si-SiNO single layer structure; (**c**) Si-W single layer structure; (**d**) initial Si-W-SiN/SiNO multilayer structure; (**e**) Si-W-SiN/SiNO multilayer structure after 1200 K annealing for 1 h.

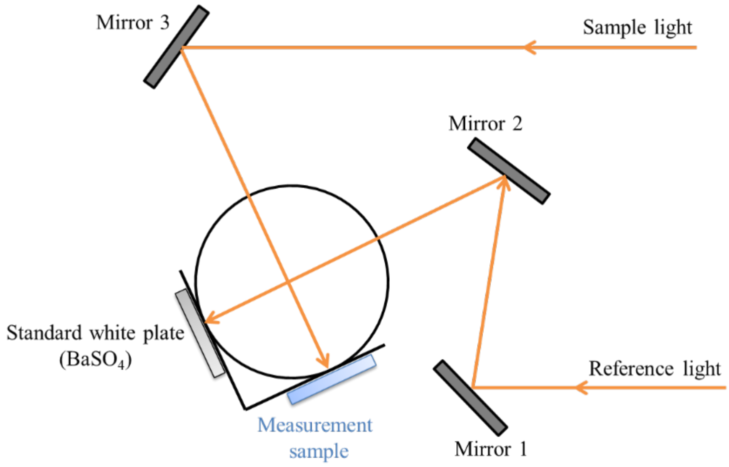

Figure S7. Optical system diagram of the integrating sphere model (ISR-3100) in UV3600 systems.

Si-W-DBR emitter with TPP resonance peak at 2 μm			
layer	SiN	SiNO	W
thickness	156 nm	305 nm	150 nm
n	2.94	1.51	1.21 @2 μm
k	0	0	7.96 @2 μm

Table S1. The thickness and refractive index of Si-W-SiN/SiNO multilayer with TPP

resonance peak at 2 μm, where n is the real part of the refractive index and k is the imaginary part of the refractive index. The dielectric constant of W is obtained from Palik's database.

	SiN	SiO	W	W-DBR-initial	W-DBR @1200 K-1 h
Rq	0.737 nm	1.93 nm	1.50 nm	2.03 nm	1.64 nm
Ra	0.582 nm	1.47 nm	1.28 nm	1.62 nm	1.25 nm
Rmax	9.64 nm	30.1 nm	10.1 nm	19.4 nm	29.5 nm

Table S2 Statistical surface roughness parameters at 5 μm scale of Si-SiN single-layer structure, Si-SiNO single-layer structure, Si-W single-layer structure, initial Si-W-SiN/SiNO multilayer structure, and Si-W-SiN/SiNO multilayer structure after 1200 K annealing for 1h. Rq means root-mean-square roughness, Ra means arithmetical average roughness, Rmax means maximum height difference.
